# Patients, doctors and the medical industry

**DOI:** 10.4103/0974-2069.64353

**Published:** 2010

**Authors:** Bharat Dalvi

Our profession continues to remain in the news – most often for the right reasons but at times for the wrong. Lately, there has been much deliberation in the print media regarding the medical industry supporting doctors for conferences, symposia, workshops and continuing medical education (CME) to gain mileage in business and boost profits.[1,2] This has prompted the Medical Council of India (MCI) to issue a notification banning doctors from accepting gifts or perquisites from the pharmaceutical or device industry.[3] In case of default, the erring physicians could face a penalty that may be as severe as losing their license for life.[4]

New drug discovery or invention of new devices requires a close collaboration between medical professionals and the medical industry. Most medical advances that have contributed to reducing morbidity and mortality are the result of such collaborative efforts. In that sense, the medical profession and the industry are partners in providing healthcare. However, their goals and guiding principles may vary. While the doctors need to keep their patients' interest at the top of their agenda, pharmaceuticals tend to put their shareholders' interest above all else.

CME is mandatory for all professionals to remain updated so that they can deliver state of the art health care to their patients. In order to conduct a good-quality CME, significant funding is necessary, which has come mainly from the industry over the past years. The industry naturally found it convenient to showcase its products and have a platform to address a large number of professionals at one shot during a mega event like annual meetings of the Cardiological or Cardiac Surgical Societies.

During the 80s and may be even early 90s, delegates met their own conference expenses while the industry made donations to the respective academic bodies at the State or the National level. This helped in subsidizing the cost to some extent. In the developed world, things started changing around this time with the medical industry lending support to individual physicians under the banner of “educational grant.” Meetings moved from normal convention centers to exotic places like beach resorts, ski slopes and luxury cruises. Some meetings had a great deal of educational content while others were merely a façade where delegates made merry, sightseeing or indulging themselves with water sports or a game of golf. India caught up with this culture rather late, but at a phenomenal speed. There were more number of meetings than the number of days in a year. Companies vied with each other to woo doctors and, most surprisingly, some of the doctors took pride in being pursued and sponsored by companies. There was another paradigm shift in the outlook of the industry toward funding of physician education. It preferred to fund individuals rather than organizations. This certainly raised the possibility of a conflict of interest while prescribing drugs or devices, since there is no “free lunch.” Various studies have demonstrated the effects of gifts and/or CME courses by the pharmaceuticals on the physician's behavior in terms of writing prescriptions.[5-8] Although there is a paucity of data on the effects of these freebies from the catheter or device companies, the results are not expected to be different. There are neurobiological mechanisms described, explaining how gifts are known to condition physicians to be favorably influenced by the companies.[9]

This prompted professional bodies like the American Medical Association (AMA), American College of Cardiology (ACC) and many other organizations in the US and other western countries to issue a whip, banning doctors from accepting favors in cash or kind from the industry. Their Indian counterpart, the MCI, has now followed suit. It certainly is a very welcome step to prevent the conflict of interest while patients are being treated. But, such a strong legislation is also expected to have some impact on training and education of the trainees or those early in their career. Many of the focused meetings and CMEs that were funded by the industry were very well planned, with a highly learned faculty and an extremely good educational content. This certainly helped the young post graduates. Given the current remuneration of post graduates, expecting them to support their travel, stay and registration may not always be economically feasible.

I think that asking the profession to divorce from the industry completely may not be a good idea, though there is no denying the fact that we need to have more transparency in the mechanism of funding by the industry. Practising doctors will need to change their mindset and pay for their education, considering it an investment that is likely to benefit them in building up their practice. Doctors-in-training could be funded through the respective departments or institutes, which, in turn could be recipients of educational grants from the industry. If the Government or the private health care sector is able to bear the burden of educating the doctors and those in training, this would probably be a great idea and then the industry could be asked to keep its hands off these activities. The absence of such an alternative will be a setback to continuing education at a time when most of the teaching institutes are dwindling in terms of quality of education. This is expected to impact the patient care as well.

So far as there is transparency in our dealings with the industry and we do not let them decide the conduct of the CME or dictate our practices, I think such honest collaborations could perhaps be acceptable. It is the current lack of openness which raises the doubts about our intentions putting us in a bad light - and very rightly so.

## References

[1] (2010). Rid the hand that heals of the muck called bribery. DNA India.

[2] Puri N (2007). Should Doctors be banned from accepting gifts from pharma companies?. Express Healthc.

[3] (2010). Times news network MCI prohibits doctors from taking gifts. The Times of India.

[4] Nagrajan R (2010). Doctors to be penalized for pharma freebies. The Times of India.

[5] Wazana A (2000). Physicians and the pharmaceutical Industry: Is a gift ever just a gift?. JAMA.

[6] Sigworth SK, Nettleman MD, Cohen GM (2001). Pharmaceutical branding of resident physicians. JAMA.

[7] Howard SM (2000). Gifts to physicians from the pharmaceutical industry. JAMA.

[8] Campbell EG, Weissman JS, Ehringhaus S, Rao SR, Moy B, Feibelmann S (2007). Institutional academic-industry relationships. JAMA.

[9] Dana J, Loewenstein G (2003). A social science perspective on gifts to physicians from industry. JAMA.

